# Small extravesicular microRNA in head and neck squamous cell carcinoma and its potential as a liquid biopsy for early detection

**DOI:** 10.1002/hed.27231

**Published:** 2022-10-22

**Authors:** Chenna R. Galiveti, Damaris Kuhnell, Jacek Biesiada, Xiang Zhang, Karl T. Kelsey, Vinita Takiar, Alice L. Tang, Trisha M. Wise‐Draper, Mario Medvedovic, Susan Kasper, Scott M. Langevin

**Affiliations:** ^1^ Division of Epidemiology, Department of Environmental & Public Health Sciences University of Cincinnati College of Medicine Cincinnati Ohio USA; ^2^ Division of Biostatistics and Bioinformatics, Department of Environmental & Public Health Sciences University of Cincinnati College of Medicine Cincinnati Ohio USA; ^3^ Division of Environmental Genetics & Molecular Toxicology, Department of Environmental & Public Health Sciences University of Cincinnati College of Medicine Cincinnati Ohio USA; ^4^ Department of Epidemiology Brown University School of Public Health Providence Rhode Island USA; ^5^ Department of Pathology & Laboratory Medicine, Alpert Medical School Brown University Providence Rhode Island USA; ^6^ Department of Radiation Oncology University of Cincinnati College of Medicine Cincinnati Ohio USA; ^7^ Cincinnati VA Medical Center Cincinnati Ohio USA; ^8^ University of Cincinnati Cancer Center Cincinnati Ohio USA; ^9^ Department of Otolaryngology University of Cincinnati College of Medicine Cincinnati Ohio USA; ^10^ Division of Hematology & Oncology, Department of Internal Medicine University of Cincinnati College of Medicine Cincinnati Ohio USA

**Keywords:** exosomes, head and neck cancer, HNSCC, HPV, miRNA

## Abstract

**Background:**

The objective was to assess secretion of small extracellular vesicular microRNA (exo‐miRNA) in head and neck squamous cell carcinoma (HNSCC) according to human papillomavirus (HPV) status, and determine the translational potential as a liquid biopsy for early detection.

**Methods:**

This study employed a combination of cell culture and case–control study design using archival pretreatment serum. Small extracellular vesicles (sEV) were isolated from conditioned culture media and human serum samples via differential ultracentrifugation. miRNA‐sequencing was performed on each sEV isolate.

**Results:**

There were clear exo‐miRNA profiles that distinguished HNSCC cell lines from nonpathologic oral epithelial control cells. While there was some overlap among profiles across all samples, there were apparent differences in exo‐miRNA profiles according to HPV‐status. Importantly, differential exo‐miRNA profiles were also apparent in serum from early‐stage HNSCC cases relative to cancer‐free controls.

**Conclusions:**

Our findings indicate that exo‐miRNA are highly dysregulated in HNSCC and support the potential of exo‐miRNA as biomarkers for HNSCC.

## 1 INTRODUCTION

The prognosis for head and neck squamous cell carcinoma (HNSCC) is relatively poor, with an overall 5‐year survival around 66% that is accompanied by a high‐degree of morbidity.^1^ While the outlook for patients with early‐stage disease is much more favorable, it worsens sharply with increasing stage at diagnosis,^2^ which is problematic since the majority of patients present at an advanced stage.^1^ Proven screening techniques for HNSCC are not currently available, aside from opportunistic visual inspection and palpation, which lacks sensitivity and varies according to the skill of the provider. The latter point is particularly important since most primary care or dental professionals at the forefront of opportunistic screening will only see a very limited number of cases over the course of their careers,^3^ underscoring the critical need for discovery and development of novel biomarkers to standardize screening and facilitate early detection.

The clinical potential of extracellular vesicles and their biomolecular cargo—including microRNA (miRNA)—as liquid‐biopsy for cancer has been well recognized in recent years.^4^, ^5^, ^6^ We previously reported extensive differential secretion of miRNA via small extracellular vesicles (sEV), which include exosomes and small microvesicles (<200 nm), by HPV‐negative (HPV−) HNSCC cell lines relative to nonpathologic oral epithelial cells.^7^ Given the increasing incidence of HPV‐positive (HPV+) HNSCC in recent years,^8^ the objective of this study was to extend our previous work to include HPV+ cell lines, thus allowing us to further assess differential secretion of sEV‐derived miRNA (exo‐miRNA), compare and contrast HPV+ with HPV− HNSCC exo‐miRNA, and determine the translational potential of exo‐miRNA as a liquid biopsy for early detection using archival serum from early‐stage HNSCC cases and cancer‐free controls.

## 2 METHODS

### 2.1 Cell lines and cell culture

For the present study, we added four HPV+ HNSCC cell lines (UPCI:SCC090, UPCI:SCC152, UM‐SCC‐47, and UM‐SCC‐104)^9^ to the four HPV− HNSCC cell lines that were previously described (FaDu, Detroit 562, Cal27, and H413).^7^ We also cultured primary human gingival epithelial cells pooled from three healthy donors (HGEPp; CELLnTEC, Bern, Switzerland) for use as nonpathologic controls. It should be noted that among the HPV+ cell lines, UPCI:SCC090 and UPCI:SCC152 are clonally related (UPCI:SCC152 is derived from a recurrence of UPCI:SCC090). HPV E6/E7 viral oncoprotein expression was confirmed via RNA in situ hybridization using the RNAscope HPV hr18 assay (Advanced Cell Diagnostics, Newark, CA), which contains probes for 18 high‐risk HPV types.^10^, ^11^, ^12^ All cell lines were authenticated by short tandem repeat (STR) fingerprinting and confirmed to be free of mycoplasma contamination using the MycoAlert™ mycoplasma detection kit (Lonza, Basel, Switzerland) prior to use.

Cells were cultivated in supplier‐recommended media with 10% fetal bovine serum (FBS), which was super‐depleted of exosomes via 18‐h ultracentrifugation at 100 000*g*, at 37°C with 5% CO_2_ in 150 cm^2^ flasks with 25 ml media. UM‐SCC‐47 and UM‐SCC‐104 were grown in DMEM with 10% FBS and nonessential amino acids. UPCI:SCC090 and UPCI:SCC152 were grown in MEM with 10% FBS, L‐glutamine, nonessential amino acids, and sodium pyruvate. HGEPp cells were grown in CnT‐PR media (CELLnTEC, Bern, Switzerland). To achieve adequate volume for sEV isolation, cells were cultured in two‐pair sets of flasks in triplicate (six flasks total per cell line). Culture media was replaced 48 h prior to cells reaching 80%–90% confluence, and media was harvested 48 h after that. Media from the paired flasks of each cell line were pooled to attain 50 ml total for sEV isolation.

sEV were isolated and purified from conditioned cell culture media (50 ml) via differential ultracentrifugation, according to the protocol described by Gallo et al.^13^ Briefly, each cell culture media sample was centrifuged at 300*g* for 10 min (4°C), followed by 2000*g* for 20 min (4°C) to remove dead cells, then at 10000*g* for another 30 min (4°C) to remove debris and large vesicles. The supernatant was then centrifuged at 100 000*g* for 70 min at 4°C on an Optima L‐100K ultracentrifuge (Beckman‐Coulter, Brea, CA). The supernatant was discarded, and the pellet was re‐suspended in 20 ml phosphate‐buffered saline (PBS) and centrifuged again at 100 000*g* for 70 min. Again, the supernatant was discarded, and the pellet was re‐suspended in 100 μl PBS and stored at −80°C for downstream analysis.

### 2.2 Human serum samples

We obtained archival pretreatment serum from the University of Cincinnati Cancer Center Biorepository from 22 incident early‐stage HNSCC cases (AJCC stage I or II) and 10 cancer‐free controls diagnosed with benign neoplasia. Cases were diagnosed at the University of Cincinnati Medical Center between 2015–2018. Controls were diagnosed within the same time frame with benign neoplastic conditions that included benign meningioma (*n* = 1), schwannoma (*n* = 2), ovarian fibrothecoma (*n* = 1), cystadenoma (*n* = 3), granuloma (*n* = 1), hamartoma (*n* = 1), and pituitary adenoma (*n* = 1). The University of Cincinnati Institutional Review Board approved the study protocol, and all subjects provided written informed consent.

Serum sEV were isolated using a previously described differential ultracentrifugation protocol.^14^ Briefly, serum (1 ml) was diluted with equal volume of PBS and centrifuged for 30 min at 2000*g* at 4°C. The supernatant was transferred to a new tube and centrifuged for 45 min at 12 000*g* at 4°C. The supernatant was collected, PBS was added to bring the total volume to 20 ml, which was gently pipetted on top of 4 ml Tris/sucrose/D_2_O solution (30% sucrose cushion) and centrifuged for 75 min at 100000*g* at 4°C with an Optima L‐100K ultracentrifuge using a 70Ti fixed‐angle rotor. A 5 ml syringe fitted with an 18‐G needle was used to collect approximately 3.5 ml of the sucrose cushion from the side of the ultracentrifuge tube. The aspirate was then transferred to a fresh ultracentrifuge tube, diluted to 60 ml with PBS, and centrifuged for 70 min at 100 000*g* at 4°C using a 45Ti fixed‐angle rotor. The sEV pellet was resuspended in 100 μl PBS and stored at −80°C for downstream analysis.

### 2.3 sEV quantitation and characterization

sEV were quantified and characterized in accordance with the minimal experimental guidelines established by the International Society for Extracellular Vesicles (ISEV).^15^

Particle size distribution and concentration was determined for each isolate via nanoparticle tracking analysis (NTA) using a NanoSight NS300 instrument (Malvern, Worcestershire, UK). sEV isolates were diluted in PBS, with the instrument set to camera level 14 and detection threshold = 5.

sEV were imaged by transmission electron microscopy (TEM) using a JEOL JEM‐1230 instrument (Tokyo, Japan). To prepare the sample, a drop of 0.1% bovine serum albumin (BSA) was placed on a formvar carbon coated grid for 1 min and subsequently removed using filter paper. Ten microliter of the sEV isolate was then placed on the grid, incubated for 5 min, and gently removed, after which 10 drops of 2% aqueous uranyl acetate (UA) were added to the grid. The UA was then removed using filter paper and the grid was allowed to dry prior to imaging.

The presence of EV‐associated tetraspanin CD81 and EV binding protein TSG101 were confirmed by Western blot.^16^ Westerns were performed for TSG101 using NuPAGE 4%–12% Bis‐Tris gels in a mini gel tank (Thermo Fisher Scientific, Waltham, MA) with MOPS SDS Running Buffer with added NuPAGE antioxidant. Samples were mixed with 4× Laemmli SDS sample buffer and NuPAGE reducing agent (10×) and heated at 95°C for 7 min. Gels were run for 50 min at 200 V constant voltage and proteins were subsequently transferred onto PVDF‐membrane using Step 1‐Transfer buffer for 7 min at 1.3A constant current on a Pierce Power Station (Thermo Fisher Scientific). Membrane was blocked for 1 h with 5% milk (CD9) or 5% BSA in 1× tris‐buffered saline containing Tween 20 (TBST). TSG101 (ab30871; Abcam, Cambridge, UK) antibody was added 1:1000 dilution in 5% milk and 5% BSA, respectively, in TBST and incubated overnight at 4°C. After washing the membrane three times for 5 min each in TBST, goat anti‐rabbit HRP secondary antibody (EXOAB‐HRP; System Biosciences) was added at 1:10000 dilution in 5% milk in TBST for 1 h and secondary antibody goat anti‐rabbit IgG H&L (ab205718, Abcam) was added at 1:2000 dilution in 5% BSA in 1× TBST for 2 h, respectively. Following three final washes for 5 min each using 1× TBST, detection was performed using Super Signal™ West Femto Maximum Sensitivity Substrate from Thermo Scientific on a ChemiDoc XRS (Bio Rad, Hercules, CA). Western blots for CD81 were performed similarly to above protocol but under nonreducing conditions (i.e., no NuPAGE antioxidant, NuPAGE reducing agent, and no sample heating to 95°C). Blocking was performed with 5% milk in 1× TBST buffer. CD81 (ab79559; Abcam) antibody was added 1:1000 dilution in 5% milk and secondary antibody goat anti‐mouse IgG H&L (ab205719; Abcam) was added 1:3000 dilution in 5% milk in TBST for 3 h.

### 2.4 microRNA sequencing

microRNA‐sequencing (miRNA‐seq) was performed by the University of Cincinnati Genomics, Epigenomics and Sequencing Core (UC GESC). Total RNA was extracted from in vitro and serum exosome isolates using the miRNeasy Micro kit (Qiagen, Valencia, CA) according to the manufacturer's recommended protocol. Total RNA concentration was estimated using a Bioanlyzer 2100 instrument with the RNA 6000 Pico kit (Agilent Technologies, Santa Clara, CA).

To prepare the library, NEBNext Multiplex Small RNA Library Prep (NEB, Ipswich, MA) was used with a modified approach. Briefly, the RNA was 3′ adaptor ligated followed by 5′ adaptor ligation, reverse transcription, and indexing via 15 cycles of PCR to generate an intact library. An approach developed by the UC GESC to process ultra‐low input and low‐quality RNA for better library yield and improved miRNA reads alignment was used for precise miRNA library size selection, ranging from 136 to 146 bp and corresponds with a 16–27 nt insert (pending in the United States (USSN 16/469242) and in Europe (17881276.4), updated on 9/8/2021). Briefly, libraries were equal‐10 μl pooled, spiked in custom designed DNA marker (135 and 146 bp) and column cleaned up. After 2.75% agarose gel electrophoresis, the miRNA library pool ranging from 135 to 146 bp including DNA marker were gel purified and quantified using NEBNext Library Quant kit (NEB) with QuantStudio 5 Real‐Time PCR system (Thermofisher, Waltham, MA). Then, the test sequencing was performed on a HiSeq sequencer (Illumina, San Diego, CA). Under single read 1 × 51 bp a few million reads were generated to quantify the relative concentration of each library. Second, based on the calculation, under the same procedure the volume of each library was adjusted to generate expected number of equal reads from each library for final data analysis.

### 2.5 Bioinformatic analysis

Demultiplexed samples were trimmed to remove adapters and retain only 16–30 base pair‐length reads and aligned to the human genome (GRCh37/hg19) using Bowtie aligner.^17^ Postalignment quality control was performed to assess miRNA transcript counts/abundance and level of replication between samples. Differential expression (DE) analysis was performed with the edgeR^18^ package for R based on miRNA transcript counts. DE of exo‐miRNA was considered to be statistically significant where false‐discovery rate (FDR)‐adjusted *p*‐values were ≤0.1.^19^ Unsupervised clustering of the 200 most variable exo‐miRNA based on coefficient of variability was performed using a Bayesian infinite mixture model.^20^ Pathway enrichment analysis of Gene Ontology (GO) Biological Process terms^21^, ^22^ was conducted via DIANA mirPath v.3^23^ for exo‐miRNA that were DE in the same direction by all eight HNSCC cell lines relative to that of oral epithelial control cells based on experimentally confirmed gene targets annotated in DIANA‐TarBase v.7.0.^24^ Enrichment *p*‐values were considered significant when FDR‐adjusted *p*‐value (*Q*) ≤ 0.05. The miRNA‐seq data has been made publicly available via deposition in the Gene Expression Omnibus (GSE84306, GSE202786, and GSE200489) (https://www.ncbi.nlm.nih.gov/geo/)

## 3 RESULTS

### 3.1 HNSCC‐derived exo‐miRNA in vitro

#### 3.1.1 Description of sEV isolates and miRNA‐seq

The mean concentration of the sEV isolates from conditioned media was 2.26 × 10^11^ particles/ml, with an average particle diameter of 143.7 nm. The particle concentration and size distribution of sEV isolates from conditioned culture media from each respective cell line are presented along with representative TEM images in Figure S1, Supporting Information. The presence of sEV‐associated tetraspanin CD81 and cytosolic protein TSG101 was confirmed via Western blot (Figure S2).

Following miRNA‐seq of the cell culture sEV isolates, the number of total sequencing reads for the HNSCC cell lines and nonpathologic oral epithelial control cells ranged from 2 367 396 to 6 175  213 (median = 3 673 186 reads), with no statistically significant differences by HPV status (*p* = 0.27) or between HNSCC and nonpathologic control cells (*p* = 0.12). Interestingly, the HPV+ cell lines had a much higher number of unique differentially secreted exo‐miRNA transcripts (336 total transcripts with an average of 239.5 differentially secreted transcripts per HPV+ cell line) compared to the HPV− cell lines (134 total transcripts with an average of 82.5 differentially secreted transcripts per HPV− cell line). In all, there were 344 unique exo‐miRNA transcripts that were differentially secreted by at least one of the eight HNSCC cell lines relative to the nonpathologic control cells (*Q* ≤ 0.1).

#### 3.1.2 Distinct exo‐miRNA profiles from HNSCC cells

**FIGURE 1:**
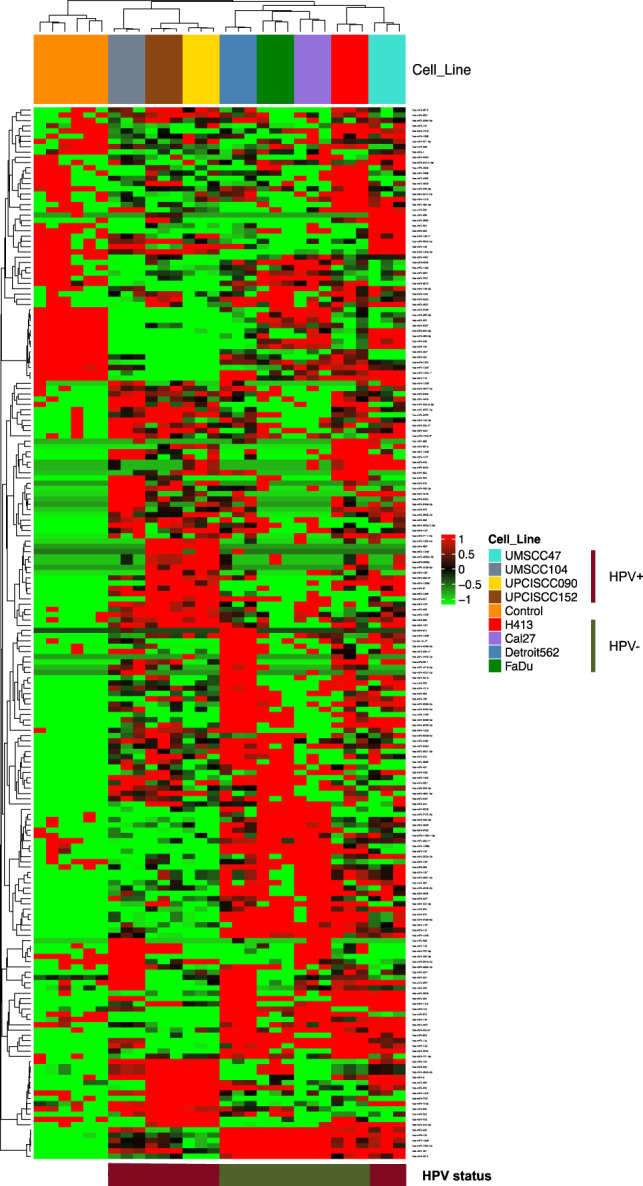
Heatmap depicting unsupervised clustering of the top 200 most variable miRNA from small extracellular vesicles (exo‐miRNA) derived from eight head and neck squamous cell carcinoma cell lines (each in triplicate) and primary nonpathologic oral epithelial control cells (six total replicates). Each column represents a unique sample and each row represents a unique exo‐miRNA transcript. The expression scale (red = high, green = low) and key corresponding to the cell lines are shown to the right of the figure [Color figure can be viewed at wileyonlinelibrary.com]

**FIGURE 2:**
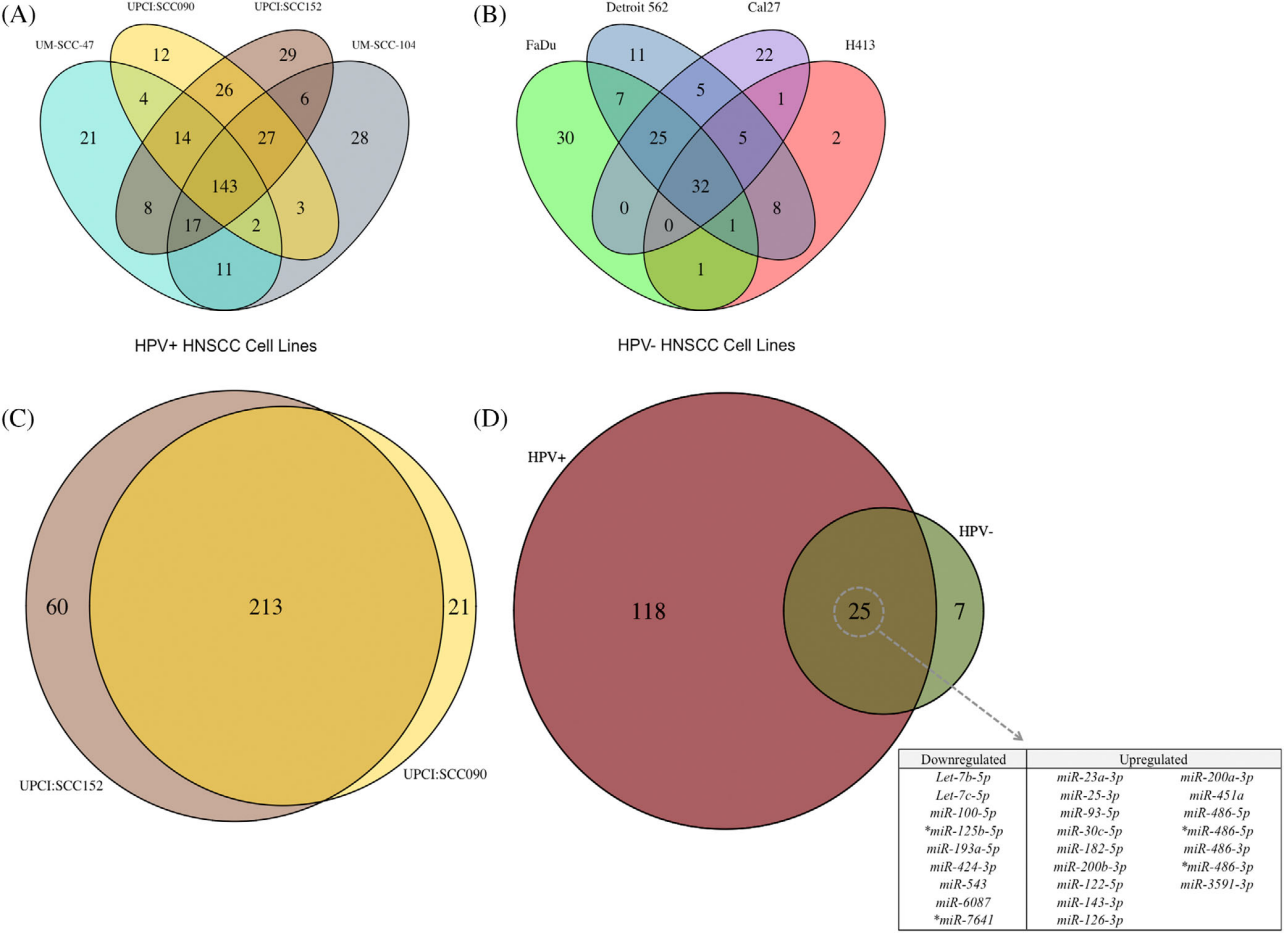
Venn diagrams of overlapping differential microRNA from small extracellular vesicles (exo‐miRNA) secreted by head and neck squamous cell carcinoma (HNSCC) cell lines relative to primary oral epithelial control cells, between (A) four HPV‐positive HNSCC cell lines; (B) four HPV‐negative HNSCC cell lines; (C) HPV‐positive HNSCC cell lines derived from an initial primary (UPCI: SCC90) and its recurrence (UPCI: SCC152); and (D) four HPV‐positive versus four HPV‐negative HNSCC cell lines, with exo‐miRNA that were commonly differentially secreted by all eight HNSCC cell lines shown at the bottom right [Color figure can be viewed at wileyonlinelibrary.com]

We observed marked differences in exo‐miRNA profiles between the HNSCC cell lines and nonpathologic oral epithelial control cells, as shown in the heatmap in Figure 1. exo‐miRNA from HNSCC cell lines clustered according to HPV‐status, with exception of the HPV+ UM‐SCC‐47 line, which segregated with the HPV− cell lines. The respective degree of overlap among HPV+ and HPV− HNSCC cell lines are shown in the Venn diagrams in Figure 2A,B. As expected, given the higher number of total differentially secreted exo‐miRNA transcripts, there was a higher degree of overlap among HPV+ cell lines, including 143 exo‐miRNA that were differentially secreted in common among all four of these lines. In contrast, there were 32 exo‐miRNA that were differentially secreted in common among all four of the HPV− cell lines. Complete lists of significantly differential exo‐miRNA for each HNSCC cell line are presented in Tables S1–S8.

It bears repeating that UPCI:SCC090 and UPCI:SCC152 are clonally related, and thus substantial overlap in exo‐miRNA was expected between them. However, this still did not explain the higher observed number of exo‐miRNA that were differentially secreted by the HPV+ HNSCC cell lines (in fact, when UPCI:SCC152 is removed from the analysis, there are 145 exo‐miRNA differentially secreted in common by the remaining three HPV+ cell lines; Figure S3).

The rationale for inclusion of the clonally related UPCI:SCC090 and UPCI:SCC152 lines was to provide contrast between a primary and recurrent HNSCC line, respectively. Indeed, there was a very high degree of overlap between these cell lines (Figure 2C), including differential secretion by the recurrent cell line (UPCI:SCC152) in the same direction of 213 of the 234 exo‐miRNA (91.0%) that were differentially secreted by the initial primary cell line (UPCI:SCC090), thus highlighting the potential utility of exo‐miRNA for monitoring recurrence during post‐treatment follow‐up. There were also 57 exo‐miRNA that were differentially secreted by the recurrent line but not by the initial primary line, along with another three exo‐miRNA that were also differentially secreted both lines but in different directions (exo‐miR‐221‐3p became upregulated in the recurrence, while exo‐miR‐205‐5p and exo‐miR‐224‐5p became downregulated).

**FIGURE 3:**
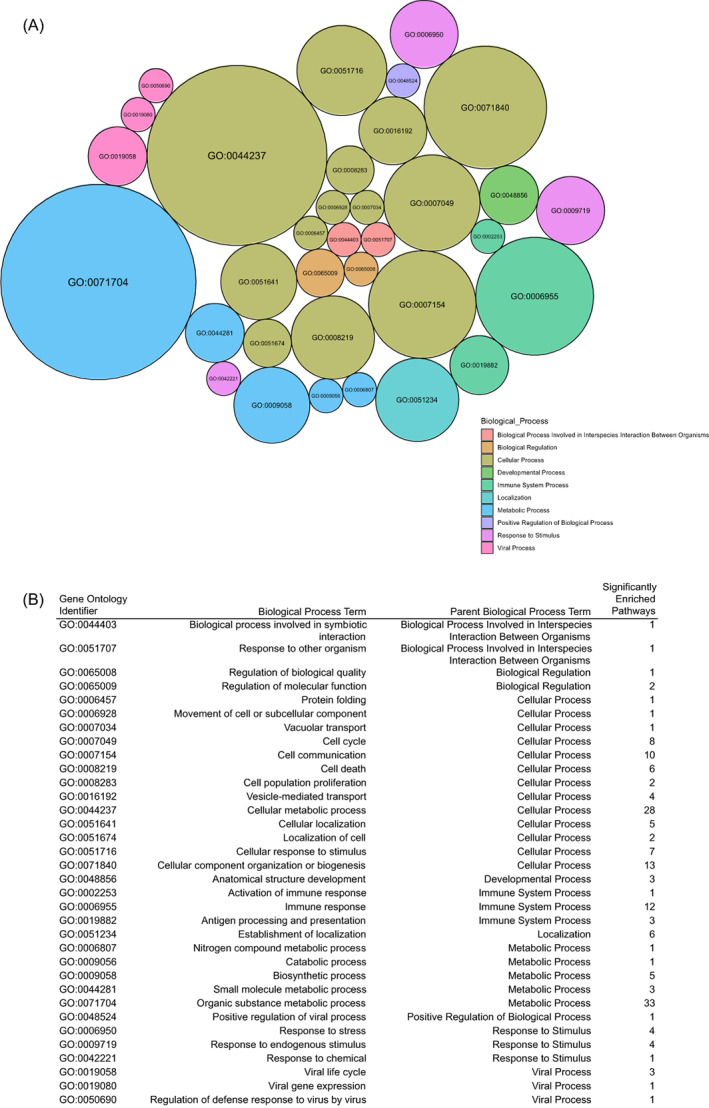
Pathway analysis for the 25 small extracellular vesicular microRNA (exo‐miRNA) that were differentially secreted in the same direction by all eight HNSCC cell lines. (A) Packed circle plot depicting overrepresented Gene Ontology (GO) Biological Processes. Circle colors correspond to the GO parent processes shown in the key at bottom right. Radii are proportional to the number of pathways associated with the respective GO term identifiers for each circle. (B) Tabular view linking the GO Identifiers in the packed circle plot to the corresponding GO Biological Processes [Color figure can be viewed at wileyonlinelibrary.com]

Further underscoring their strong potential as HNSCC biomarkers, there were 25 exo‐miRNA that were universally differentially secreted by all eight HNSCC cell lines relative to the nonpathologic oral epithelial control cells, including nine that were downregulated and 16 upregulated (Figure 2D). Pathway analysis of these 25 exo‐miRNA revealed overrepresentation of numerous pathways (Figure 3 and Table S9), in particular (although not limited to) those relating to organic substance metabolic process (GO:0071704), cellular metabolic process (GO:0044237), cellular component organization or biogenesis (GO:0071840), immune response (GO:0006955), and cell communication (GO:0007154).

### 3.2 Differential secretion of exo‐miRNA by cases with early‐stage HNSCC

#### 3.2.1 Study population

To further assess the potential of exo‐miRNA as biomarkers of HNSCC, we isolated sEV from pretreatment serum samples from cases diagnosed with early stage HNSCC (*n* = 22; AJCC stage I/II) and cancer‐free controls diagnosed with various benign neoplastic conditions (*n* = 10). HNSCC cases were somewhat older and more likely to be male compared to controls, although these differences were not statistically significant (*p* = 0.09 and 0.10, respectively). A full description of demographic and clinical characteristics of cases and controls is presented in Table 1.

**TABLE 1:** Description of early‐stage head and neck squamous cell carcinoma cases (HNSCC) and cancer‐free controls diagnosed with benign neoplasia

	HNSCC cases (*n* = 22)	Benign controls^a^ (*n* = 10)	*p*_difference_
Age, mean years (*σ*)	64 (10.9)	55 (14.1)	0.09^c^
Sex, *n* (%)			
Female	4 (18%)	5 (50%)	0.10^d^
Male	18 (82%)	5 (50%)	
Race, *n* (%)			
Black	1 (5%)	2 (20%)	0.22^d^
White	21 (95%)	8 (80%)	
Smoking, *n* (%)			
Never	5 (23%)	3 (30%)	>0.99^d^
Former	10 (45%)	4 (40%)	
Current	7 (32%)	3 (30%)	
Pack‐years^b^, mean years (*σ*)	45 (22.3)	49 (22.3)	0.75 ^e^
Primary tumor site, *n* (%)			
Oral cavity	14 (64%)		
Oropharynx	4 (18%)		
Larynx	4 (18%)		
AJCC TNM stage group, *n* (%)			
I	7 (32%)		
II	15 (68%)		
Histologic grade, *n* (%)			
Well differentiated (G1)	5 (29%)		
Moderately differentiated (G2)	8 (47%)		
Poorly differentiated (G3)	4 (24%)		
p16 immunohistochemistry			
Positive	3 (14%)		
Negative	6 (17%)		
Unknown/not tested	13 (59%)		

Abbreviations: AJCC, American Joint Committee on Cancer; HNSCC, head and neck squamous cell carcinoma; TNM, tumor, node, metastasis; *σ*, standard deviation.

^a^ Meningioma (1), schwannoma (2), ovarian fibrothecoma (1), cystadenoma (3), granuloma (1), hamartoma (1), pituitary adenoma.

^b^ Current and former smokers only.

^c^ Two sample *t* test with Satterthwaite's correction for unequal variance.

^d^ Fisher's exact test.

^e^ Two‐sample *t* test.

The average concentration was 7.13 × 10^9^ particles/ml (mean particle size = 120.0 nm diameter) for the random sample of early‐stage cases (*n* = 3) and 1.48 × 10^10^ particles/ml (mean particle size = 134.8 nm diameter) for the random sample of cancer‐free controls (*n* = 3). The number of total sequencing reads for serum sEV isolates ranged from 179 675 to 1 535  750, with a median = 692 055 reads.

**FIGURE 4:**
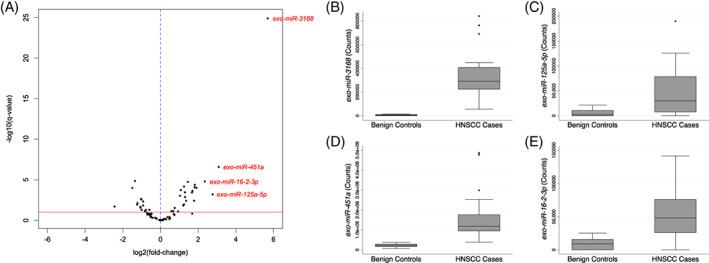
Differentially secreted small extracellular vesicular microRNA (exo‐miRNA) in pretreatment serum of early‐stage head and neck squamous cell carcinoma (HNSCC) cases versus cancer‐free controls with benign neoplastic conditions: (A) Volcano plot showing differential secretion of exo‐miRNA for early‐stage cases relative to controls. The horizontal red line corresponds *Q* = 0.1 and the vertical dashed line corresponds to log2 fold‐change = 0. The box plots on the right show the respective distribution of exo‐miRNA for early‐stage cases and benign controls for (B) exo‐miR‐3168; (C) exo‐miR‐125a‐5p; (D) exo‐miR‐451a; and (E) exo‐miR‐16‐2‐3p [Color figure can be viewed at wileyonlinelibrary.com]

A total of 41 mature exo‐miRNA were differentially present in serum from early‐stage HNSCC cases relative to the benign neoplasia samples (*Q* < 0.1; Figure 4A). Of these, 17 were present in lower quantities in serum of cases, with fold‐change ranging from 0.18 to 0.66, while 24 were oversecreted with fold‐change ranging from 1.49 up to 51.72. Notably, 20 of the 24 oversecreted exo‐miRNA were present at >2‐fold change higher, underscoring the translational potential as biomarkers for diagnosis or early detection of HNSCC. The complete list of differential serum exo‐miRNA is available in Table S10.

When we examined the distribution of expression levels for each of the top four most highly oversecreted exo‐miRNA, the expression for all control samples fell below the median levels observed in early stage HNSCC cases (Figure 4). Strikingly, secretion levels of exo‐miR‐3168 and exo‐miR‐451a in control samples were below what was detected for any of the HNSCC cases. There were no significant correlations between the top four overexpressed exo‐miRNA and key clinical and demographic characteristics (Table 2), with the exception of exo‐miR‐451a, which exhibited only fair/modest correlation^25^ with the primary tumor site (*ρ* = 0.45, *p* = 0.04), thus highlighting their relative stability as HNSCC biomarkers.

**TABLE 2:** Correlation of top four oversecreted small extracellular vesicular microRNA (exo‐miRNA) levels with sociodemographic and clinical characteristics of early‐stage head and neck squamous cell carcinoma cases

	exo‐miR‐3168		exo‐miR‐451a		exo‐miR‐125a‐5p		exo‐miR‐16‐2‐3p	
	*ρ*	*p*‐value	*ρ*	*p*‐value	*ρ*	*p*‐value	*ρ*	*p*‐value
Age	−0.16	0.46	0.25	0.25	−0.08	0.73	0.30	0.18
Sex	−0.04	0.87	0.35	0.11	0.11	0.62	0.07	0.74
Smoking (never, former, current)	−0.13	0.56	−0.15	0.51	0.03	0.88	−0.25	0.26
Alcohol consumer (yes or no)	−0.31	0.16	−0.21	0.35	−0.02	0.92	−0.18	0.42
Primary tumor site	−0.13	0.56	**0.45**	**0.04**	−0.03	0.89	−0.05	0.83
AJCC TNM stage group (I vs. II)	0.25	0.25	−0.05	0.81	0.26	0.25	−0.07	0.76
Tumor grade	−0.04	0.88	−0.12	0.64	−0.11	0.68	−0.24	0.36
p16 immunohistochemistry	−0.18	0.64	−0.18	0.64	0.00	>0.99	0.00	>0.99

*Note*: Bold text denotes significant value (*p* < 0.05).

Abbreviations: AJCC, American Joint Committee on Cancer; TNM, tumor, node, metastasis; *ρ*, Spearman's rank correlation coefficient.

Lending further strength of evidence, exo‐miR‐451a (8.52 fold‐change higher in serum of cases) was also significantly oversecreted by all eight of the HNSCC cell lines relative to nonpathologic oral epithelial control cells; *e*xo‐miR‐16‐2‐3p (5.10 fold‐change higher in serum of cases) was similarly significantly oversecreted by seven of the eight HNSCC cell lines. Conversely, however, exo‐miR‐3168 was downregulated by all four HPV− lines and not detected in the conditioned media from the HPV+ lines, while exo‐miR‐125a‐5p was downregulated in all four HPV+ lines and not detected in media from the HPV− lines, which may suggest that the high levels observed in blood were derived from other components of the tumor microenvironment or some other systemic response to the HNSCC tumor cells.

## 4 DISCUSSION

Our results demonstrate distinct exo‐miRNA secretion patterns by HNSCC, both in vitro and in human serum, and highlight the strong potential of exo‐miRNA for use as biomarkers for early detection or diagnosis. While notable differences were observed according to HPV status, there were also a number of differentially secreted exo‐miRNA that transcended across all cancers, regardless of HPV status, some of which were markedly higher in serum of early stage HNSCC cases relative to cancer‐free controls.

While there has been a lot of speculation by researchers on the putative value of exo‐miRNA as biomarkers of HNSCC,^26^ the role of circulating exo‐miRNA has remained considerably understudied to date. Only a couple of studies have attempted to comprehensively assess differential secretion of exo‐miRNA, or exo‐miRNA in plasma or serum^27^, ^28^: one of which did not include any early‐stage cases,^27^ whereas the remaining study of serum exosomes by Zhao and colleagues included 50 laryngeal squamous cell carcinoma cases (LSCC; including 31 early‐stage cases).^28^ While there were differences in serum exo‐miRNA identified in our study relative to the other two studies in the literature,^27^, ^28^ which is not surprising given the differences in study design and restriction to larynx and oropharynx, respectively, there were also some similarities. Notably, exo‐miR‐27a was identified by all three studies, with similar overexpression in our study (2.73‐fold increase) and Zhao et al. (2.18‐fold increase)^26^; exo‐miR‐125a (upregulated) and exo‐miR‐320a (downregulated) were also dysregulated in the same direction in both, highlighting the potential for reproducible and generalizable results.

Two of the top four exo‐miRNA that were differentially present at higher levels in serum of early‐stage HNSCC cases relative to cancer‐free controls were also differentially overexpressed by the HNSCC cell lines. The first, exo‐miR‐451a, was oversecreted by all eight HNSCC cell lines compared to oral epithelial control cells. This appeared to be independent of HPV status since four of the HNSCC lines were HPV− and the other four were HPV+. At the time of submission, 27 experimentally confirmed mRNA targets were catalogued in a database of miRNA targets (TarBase v7.0) including multiple genes in the mTOR signaling pathway (KEGG: hsa04150), for example, exo‐miR‐451a, which has been reported to be upregulated in serum or plasma from other aerodigestive epithelial cancers.^29^, ^30^, ^31^ The second, exo‐miR‐16‐2‐3p, was differentially oversecreted by seven of the eight HNSCC cell lines included in our study, again implying that this was independent of HPV‐status. The functional significance of exo‐miR‐16‐2‐3p oversecretion is complicated, given that miR‐16‐2‐3p has 2886 experimentally confirmed targets (TarBase v7.0). It is a known tumor suppressor miRNA with targets that include key oncogenes in pathways in cancer (KEGG: hsa05200), and elevated exo‐miR‐16‐2‐3p has been associated with papillary thyroid carcinoma.^32^ Conversely, exo‐miR‐3168 and exo‐miR‐125a‐5p were either downregulated or not detected in conditioned media from the HNSCC cell lines that we interrogated. The significance of this is unclear, although it is worth noting that serum sEV do not solely contain HNSCC‐derived sEV but rather a heterogeneous mix of sEV from a wide variety of cell types. Thus it is possible that the observed high levels of these exo‐miRNA are attributable to a systemic response to the cancer cells or from nonmalignant cells within the tumor microenvironment. The exact function or contribution of exo‐miRNA in carcinogenesis can be complex given the large number of mRNA targets for each miRNA. To this point, miR‐125a‐5p, a known tumor suppressor miRNA for multiple cancer types,^33^, ^34^, ^35^, ^36^, ^37^ including HNSCC,^38^, ^39^ has more than 900 experimentally confirmed targets (TarBase v7.0), which include numerous cancer‐associated pathways. To our knowledge, at the time of submission, miR‐3168 had no experimentally confirmed targets. However, a recent study reported that cell‐free miR‐3168 in plasma from HNSCC patients was a predictor of cisplatin‐induced nephrotoxicity in HNSCC.^40^

The finding of altered exo‐miRNA secreted by HPV+ HNSCC cells in vitro expands on our previous findings of HNSCC‐specific exo‐miRNA profiles in HPV− HNSCC cells^7^ and further strengthens the evidence for extensive dysregulation exo‐miRNA across HNSCC. Our observed HNSCC‐specific exo‐miRNA secretion profiles are in‐line with those of a recent study by Masaoka and colleagues,^41^ who reported that multiple exo‐miRNA were commonly differentially secreted by oral squamous cell carcinoma cell lines relative to nonpathologic oral keratinocytes, including universal downregulation of exo‐miR‐125b‐5p and universal upregulation of exo‐miR‐23a‐3p, which were each differentially secreted in the same respective direction by all eight of the HNSCC cell lines in our study.

Although we observed overlap of differential exo‐miRNA across HNSCC cell lines, there were apparent differential profiles between HPV+ and HPV− HNSCC. HPV+ HNSCC cell lines had a higher number of differentially secreted exo‐miRNA compared with HPV− HNSCC cell lines, and HNSCC cell lines segregated according to HPV status during unsupervised cluster analysis, with the exception of the HPV+ cell line UM‐SCC‐47, which clustered with the HPV− cell lines. While we confirmed expression of high‐risk HPV viral oncogenes by UM‐SCC‐47, it should be noted that researchers at the University of Michigan observed weak p16 expression^42^ and the lowest relative E6 and E7 expression of the four HPV+ cell lines that were included our study.^9^ The observed differences in exo‐miRNA profiles for HPV+ versus HPV− HNSCC cells is consistent with results reported by others.^43^, ^44^

Many of the pathways overrepresented among exo‐miRNA that were commonly differentially secreted by all HNSCC cell lines impact known hallmarks of cancer.^45^ Notably, these include pathways known to be perturbed in HNSCC^46^ relating to immune evasion, including four pathways involving to MHC class I or II antigen processing or presentation; tumor‐promoting inflammation, including pathways related to toll‐like receptors; sustained proliferative signaling; replicative immortality; and reprogramming energy metabolism, including two hypoxia‐related pathways.

The extensive overlap in exo‐miRNA between the two cell lines derived from a primary (UPCI:SCC090) and recurrent tumor (UPCI:SCC152) from the same patient is of potential importance in terms of application of exo‐miRNA profiles from the initial primary for post‐treatment follow‐up surveillance. Greater than 90% of the exo‐miRNA that were differentially secreted by the cell line derived from the initial primary were also differentially secreted in the same direction by that derived from its recurrence. However, given the emerging role of extracellular vesicles in cancer progression and remodeling of the tumor microenvironment,^47^ the subtle differences in exo‐miRNA profiles between these two cell lines may also contain critical information regarding the biology of recurrence, suggesting a need for additional downstream functional studies.

The significance of the differential expression of exo‐miR is unclear, although it is worth noting that serum sEV do not solely contain HNSCC‐derived sEV, but also a heterogeneous mix of sEV derived from a wide variety of cell types. Thus it is possible that the observed high levels of these exo‐miRNA are due to a systemic response to the cancer cells, or even from nonmalignant cells, within the tumor microenvironment. The exact function or contribution of exo‐miRNA in carcinogenesis is also complex given the large number of mRNA targets for each miRNA. Taken together, these observations emphasize the urgent need to determine their function, their potential as biomarkers for diagnosis or monitoring disease recurrence, or even their potential as targets for therapy. Incorporation of exo‐miRNA as correlative biomarkers for monitoring disease in future clinical trials would likely provide necessary insight on the potential of exo‐miRNA as predictive and prognostic biomarkers.

Notable strengths of the present study include interrogation of a diverse set of both HPV+ and HPV− HNSCC cell lines, comprehensive miRNA‐sequencing of exo‐miRNA, and availability of serum collected prior to initiation of treatment for cases with incident early‐stage HNSCC. Further, by drawing a comparison to cancer‐free control subjects diagnosed with variety of nonmalignant lesions, we are able to demonstrate the clinical potential of exo‐miRNA to distinguish cases with early‐stage HNSCC from those with benign neoplasia. However, there were also some limitations to our study. One potential limitation was our use of two‐dimensional cell culture, which may not accurately recapitulate the secretome of the tumor microenvironment. However, the tradeoff was that this allowed us to isolate pure populations of HNSCC‐derived sEV, permitting us to better determine cancer‐associated alterations. Further, while the availability of pre‐treatment serum from early‐stage HNSCC cases was a strength, we only interrogated serum sEV from a modest number of cases and controls. While our results underscore the strong translational potential of circulation exo‐miRNA for early detection of HNSCC, our results require additional prospective validation in an independent cohort to better determine their clinical utility.

## 5 CONCLUSIONS

In summary, our findings indicate that exo‐miRNA are highly dysregulated in HNSCC and may contribute to acquisition of hallmarks of cancer during the carcinogenic process, which warrants additional downstream study to assess the potential as therapeutic targets. Importantly, our findings also support the great clinical potential of exo‐miRNA as biomarkers for HNSCC. Continued discovery and development of novel biomarkers that facilitate early detection of incident and recurrent/new primary HNSCC is of paramount importance in terms of reducing the high level of morbidity and mortality that stems from this devastating disease.

## CONFLICT OF INTEREST

The authors declare that there is no conflict of interest that could be perceived as prejudicing the impartiality of the research reported.

## Supporting information


**Appendix S1** Supporting Information.

## Data Availability

The miRNA‐seq data have been made publicly available via deposition in the Gene Expression Omnibus (GSE84306, GSE202786, and GSE200489): https://www.ncbi.nlm.nih.gov/geo/.
